# Drought stress prediction and propagation using time series modeling on multimodal plant image sequences

**DOI:** 10.3389/fpls.2023.1003150

**Published:** 2023-02-09

**Authors:** Sruti Das Choudhury, Sinjoy Saha, Ashok Samal, Anastasios Mazis, Tala Awada

**Affiliations:** ^1^ School of Natural Resources, University of Nebraska-Lincoln, Lincoln, NE, United States; ^2^ School of Computing, University of Nebraska-Lincoln, Lincoln, NE, United States; ^3^ Institute of Radio Physics and Electronics, University of Calcutta, Kolkata, West Bengal, India; ^4^ Department of Civil and Environmental Engineering, University of California, Merced, Merced, CA, United States; ^5^ Agricultural Research Division, University of Nebraska-Lincoln, Lincoln, NE, United States

**Keywords:** stress prediction, image sequence analysis, time series modeling, dynamic time warping, temporal stress propagation, spectral band difference segmentation, deep neural networks

## Abstract

The paper introduces two novel algorithms for predicting and propagating drought stress in plants using image sequences captured by cameras in two modalities, i.e., visible light and hyperspectral. The first algorithm, VisStressPredict, computes a time series of holistic phenotypes, e.g., height, biomass, and size, by analyzing image sequences captured by a visible light camera at discrete time intervals and then adapts dynamic time warping (DTW), a technique for measuring similarity between temporal sequences for dynamic phenotypic analysis, to predict the onset of drought stress. The second algorithm, HyperStressPropagateNet, leverages a deep neural network for temporal stress propagation using hyperspectral imagery. It uses a convolutional neural network to classify the reflectance spectra at individual pixels as either stressed or unstressed to determine the temporal propagation of stress in the plant. A very high correlation between the soil water content, and the percentage of the plant under stress as computed by HyperStressPropagateNet on a given day demonstrates its efficacy. Although VisStressPredict and HyperStressPropagateNet fundamentally differ in their goals and hence in the input image sequences and underlying approaches, the onset of stress as predicted by stress factor curves computed by VisStressPredict correlates extremely well with the day of appearance of stress pixels in the plants as computed by HyperStressPropagateNet. The two algorithms are evaluated on a dataset of image sequences of cotton plants captured in a high throughput plant phenotyping platform. The algorithms may be generalized to any plant species to study the effect of abiotic stresses on sustainable agriculture practices.

## 1 Introduction

Increasing demands for food, fuel, fiber, and feed to meet the needs of the growing population, under climate change, and dwindling natural resources constitute a major challenge confronting sustainable agriculture in the 21st century. In addition, climate change has affected the intensity and frequency of drought and extreme weather events in many regions, increasing food insecurity and affecting the livelihoods of many communities (Sheffield et al., 2012; Ort et al., 2015). In fact, it is estimated that about two thirds of crop losses in the last half century were caused by drought (Comas et al., 2013). Thus, an improved understanding of the plant’s response to increased water stress as a function of time is an important step in shepherding breeding efforts, developing smart agricultural practices, and enhancing the decision making process to mitigate and adapt to climate change.

A time series is an ordered sequence of values of a variable measured at successive points in time, often at regular time intervals, e.g., weather forecasts, stock prices, biometrics, and exchange rates in finance. Based on the variable, a time series can be classified as either continuous or discrete. In the case of a continuous time series, observations are measured continuously over time, e.g., temperature readings, and the flow of a river. On the other hand, a discrete time series is characterized by recordings at typically equally spaced time intervals, e.g., daily, weekly, or yearly. High throughput plant phenotyping (HTPP) refers to the imaging of plants captured at regular intervals for a significant time period to extract the salient information about a plant’s development and metabolism that are manifested at different wavelengths of the electromagnetic spectrum. Visible light image sequences are used to extract morphological characteristics of the plants or their organs (Dyrmann, 2015; Das Choudhury et al., 2018). In contrast, infrared images can serve as a proxy for a plant’s temperature, which in turn can be used to detect differences in stomatal conductance, a measure of a plant’s response to water status and transpiration rate for abiotic stress adaptation (Li et al., 2014). Hyperspectral cameras typically capture a scene in hundreds of bands covering a broad range of wavelengths at very narrow intervals. Since hyperspectral imaging has the highest coverage of the electromagnetic spectrum, it has the potential for a wide variety of applications, including the detection of abiotic and biotic stresses in plants and the measurements of chlorophyll content, canopy senescence, and water content (Gampa and Quinones, 2020). In this paper, we used time-series image sequences captured by two types of cameras, i.e., visible light and hyperspectral, for stress prediction and temporal stress propagation.

The images in an HTPP platform are captured at regular intervals with timestamps to compute phenotypes, i.e., the observable traits of plants as a result of the complex interaction between genotype and environment. Imaging at regular intervals facilitates the extraction of smart phenotypic traits, e.g., predicting the onset of stress and its temporal propagation patterns in a plant. Since the process of phenotypic trait extraction based on image analysis is nondestructive in nature, the traits may be extracted at multiple timestamps in a plant’s life cycle. The phenotypes computed by analyzing the images captured in an HTPP may be modeled as a discrete time series. These abstractions and subsequent computations are not possible from manual measurements.

The phenotypic time series can be classified into the following four categories (Das Choudhury, 2020b):

- *Nonlinear*: A phenotypic time series that tends to increase, decrease or stagnate over time is referred to as a nonlinear time series. The total leaf area of a plant increases over time under normal growth conditions, however, it often starts to decrease as some leaves experience curling or shedding due to exposure to stress, e.g., drought, thermal, and salinity. Note that for many cereal crops, e.g., maize and sorghum, the plant height increases monotonically with time and then remains stagnant upon completion of the vegetative stage.
- *Recovery*: The normal growth of a plant is significantly affected under stress. However, if the stress condition is reverted, i.e., re-watering of a drought-stressed plant or adjusting the temperature of a plant under thermal stress, normal growth may resume under certain circumstances.
- *Seasonal*: Plants undergo internal physiological seasonal changes leading to changing leaf colors, shedding, blooming, and generating new leaves. A time series representing the total number of leaves over a growing season can demonstrate this effect.
- *Catastrophic*: A catastrophic phenotypic time series reflects any significant impact on a plant’s development due to unprecedented events, e.g., floods, storms, and earthquakes, and hence does not follow any specific pattern.

This paper presents two algorithms to understand the dynamics of stress in plants from image sequences. It first describes a predictive model to determine if a plant is under stress, using the time series of holistic phenotypes or traits computed by analyzing visible light image sequences using dynamic time warping (DTW) - a statistical method extensively used to analyze temporal sequences, including applications in speech recognition and biometric verification (Das Choudhury and Tjahjadi, 2013). The paper introduces a novel dynamically growing subsequence based DTW matching algorithm for stress prediction using the phenotypic time series.

Deep neural networks have been successfully employed in high throughput temporal plant phenotyping for a variety of applications (Bashyam et al., 2021; Zheng et al., 2021; Das Choudhury et al., 2022). The method in (Das Choudhury et al., 2022) performs automated flower detection from multi-view image sequences to determine a set of novel phenotypes, e.g., the emergence time of the first flower, the total number of flowers present in the plant at a given time, flower growth trajectory, and blooming trajectory. A graph theoretic approach has been used by (Bashyam et al., 2021) to detect and track individual leaves of a maize plant for automated growth stage monitoring. The method by (Azimi et al., 2021) uses Convolutional Neural Network - Long Short Term Memory (CNN-LSTM) for water stress classification in chickpea plants, whereas the method by (Taha et al., 2022) uses deep convolutional neural networks (DCNNs) to diagnose the nutrient status of lettuce grown in aquaponics. In this paper, we present a novel algorithm based on convolutional neural networks to determine the qualitative and quantitative propagation of drought stress in cotton plants by classifying reflectance spectra generated from hyperspectral image sequences.

The efficacy of the two algorithms is demonstrated using a set of cotton (*Gossypium* spp.) plants. Cotton, a C3 crop known for its valued fiber (cotton lint), supplies about 79% of the global natural fiber used in the textile industry (Dabbert and Gore, 2014; Townsend and Sette, 2016), while its seeds provide nutrition to both humans and animals (Bertrand et al., 2005). Drought stress has been identified as a major impediment to cotton production. In cotton, drought stress causes a reduction in both quantity and quality of lint (Pettigrew, 2004; Wang et al., 2016) with a severe negative impact on a farmer’s income and supply of raw material for the textile industry. Although the algorithms introduced in this paper are evaluated on a cotton dataset, they are generic and, thus, are applicable to any plant species subjected to any kind of stress, i.e., drought, salinity, and thermal, to quantitatively determine the impact of stress as a function of time.

## 2 Materials and methods

In this section, we first describe the dataset used to develop and evaluate the two algorithms, i.e., VisStressPredict and HyperStressPropagateNet, followed by the detailed descriptions of these algorithms.

### 2.1 Dataset

The image sequences used for algorithm development and evaluation were obtained at the Innovation Campus greenhouse of the University of Nebraska-Lincoln (Lincoln, Nebraska, U.S.) using High Throughput Plant Phenotyping Core Facilities (HTPP, Scanalyzer 3D, LemnaTec Gmbh, Aachen, Germany). Chemically-delinted black cotton seeds (variety PHY 499 WRF) were planted in 5.7 L pots (22 cm diameter and 20 cm height) filled with 25% sand and 75% standard greenhouse mix, at approximately 24°C, and RH 58%. The daytime Photosynthetic Active Radiation (PAR) was supplemented with LED red/blue light, with an intensity of 200 *μmol m* ^-2^ *s* ^-1^. The photoperiod in the greenhouse was set at 17 hours throughout the study to standardize the light conditions. After germination, plants were maintained on the bench where nutrients and water were applied following a standard greenhouse management regime. After two weeks, plants were randomly divided into two groups of 10 corresponding to the two experimental groups (i.e., Experiments 1 and 2). Each experimental group was further split into two groups of 5 plants and assigned to treatment groups (control and drought stress). The onset of the dry-down and the duration of the experiment varied across the two experiments. In Experiment 1, dry-down was initiated 12 days after the onset of plant imaging and lasted for 8 days. A week later, a similar dry-down was initiated for the second experimental group and lasted for 9 days.

Each plant was placed in a metallic carrier (dimension: 236 mm × 236 mm × 142 mm) on an automated conveyor belt that moves the plants from the greenhouse to the four imaging chambers successively for capturing images in different modalities. It has three watering stations with a balance that can add water to a target weight or specific volume and records the specific quantity of water added daily. The images of the greenhouse with plants placed on the automated conveyor belt, the watering station, and plants entering into the imaging cabinets are available in (Das Choudhury et al., 2018; Das Choudhury et al., 2022). The cameras installed in the four imaging chambers are (a) visible light - side view and top view (Prosilica GT6600 29 megapixel camera with a Gigabit Ethernet interface ^1^), (b) infrared - side view and top view (Pearleye p-030 LWIR), (c) fluorescent - side view and top view (Basler Scout scA1400-17gm/gc), and (d) hyperspectral - side view (Headwall Hyperspec Inspector x-vnir ^2^) and near-infrared - top view (Goldeye p-008 SWIR), respectively. Each imaging chamber has a rotating lifter for up to 360 side view images. In this study, we used visible light images (captured from five side-views, i.e., 0°, 72°, 144°, 216°, 288°) for VisStressPredict algorithm and hyperspectral images for HyperStressPropagateNet algorithm. The average time interval between a plant entering into and exiting from each of the first three imaging chambers for capturing five side view images is approximately 1 minute and 10 seconds. Since a hyperspectral camera typically captures a scene in hundreds of bands at a narrow interval over a broad range of the spectrum, its image capturing time is significantly higher than that of the other imaging modalities. In our HTPP facility, the time to capture a single side view image of a plant using a hyperspectral camera (total number of bands: 243; spectrum range: 546 nm to 1700 nm) is approximately 2 minutes and 15 seconds. All images are exported as PNG file types. Pots were automatically weighed upon exiting the hyperspectral chamber, and water was applied daily to designated levels to reach a predetermined percentage of field capacity (50%). **Table 1** provides detailed information on the specifications of the cameras of our HTPP system.

**Table 1:** Camera specifications of the HTPP system at the UNL, USA.

Image type	Camera sensor	Spectral range (nm)	Spatial resolution	Bit depth	Frame rate
Visible light	AVT Prosilica GT6600	400-700	6576 × 4384	14 (mono) - 12 (color)	4
Fluorescent	Basler Scout scA1400-17GC	620-900	1390 × 1038	12	17
Near infrared	Goldeye P-008 SWIR	900-1700	320 × 256	12	118
Infrared	Pearleye P-030 LWIR	800-1400	640 × 480	14	24
Hyperspectral	Headwall Hyperspec Inspector X-VNIR	546-1700	320 × 561	8	–

### 2.2 VisStressPredict: DTW based stress prediction using visible light imagery

#### 2.2.1 Image-based phenotypic time series computation

**Figure 1:**
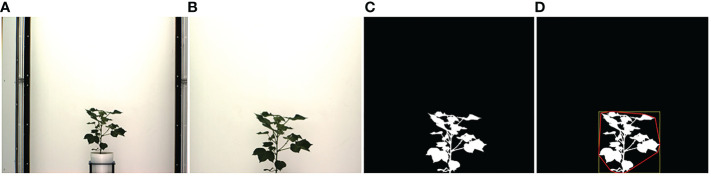
Illustration of holistic phenotype computation based on image analysis: **(A)** original image; **(B)** cropped image; **(C)** binary image; **(D)** plant enclosed by bounding rectangle and convex hull.

In this section, we describe the steps to compute phenotypic time series based on analyzing image sequences. Visible light images are used to compute structural phenotypes that characterize a plant's morphology. Image-based structural phenotypes can either be computed by considering the whole plant as a single object (holistic phenotypes) or by considering individual components of the plants, e.g., stem, leaves, fruits, and flowers (component phenotypes). **Figure 1** shows the intermediate images in the computation of three holistic structural phenotypes, i.e., the height of the plant, the area of the convex hull enclosing the plant, and the total number of plant pixels, all of which contribute to the measurement of plant growth and development. First, the original plant image sequences are cropped to a fixed size to remove the frames of the imaging cabinet and the pot. **Figure 1A** shows a sample original image, and **Figure 1B** shows the corresponding cropped image that retains the plant. The cropped image is then binarized in the LAB color space using color thresholding (**Figure 1C**). Finally, the binary image thus obtained is enclosed by primitive geometric shapes, e.g., bounding rectangle and convex hull (**Figure 1D**), to compute holistic phenotypes.

**Figure 2:**
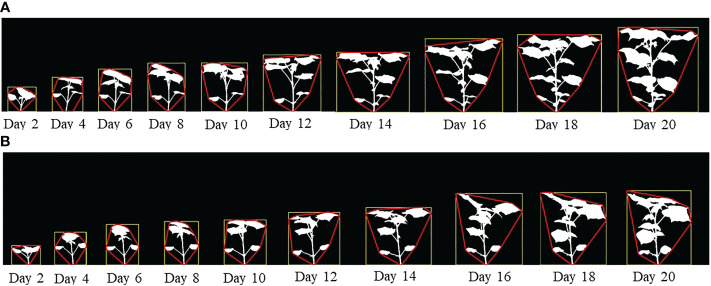
**(A)** An image sequence of a sample plant for side view angle of 0° (Experiment 1) enclosed by their bounding rectangles and convex hulls under controlled condition; and **(B)** An image sequence of a sample plant for side view angle of 0° (Experiment 1) enclosed by their bounding rectangles and convex hulls subjected to drought stress.

**Figure 3:**
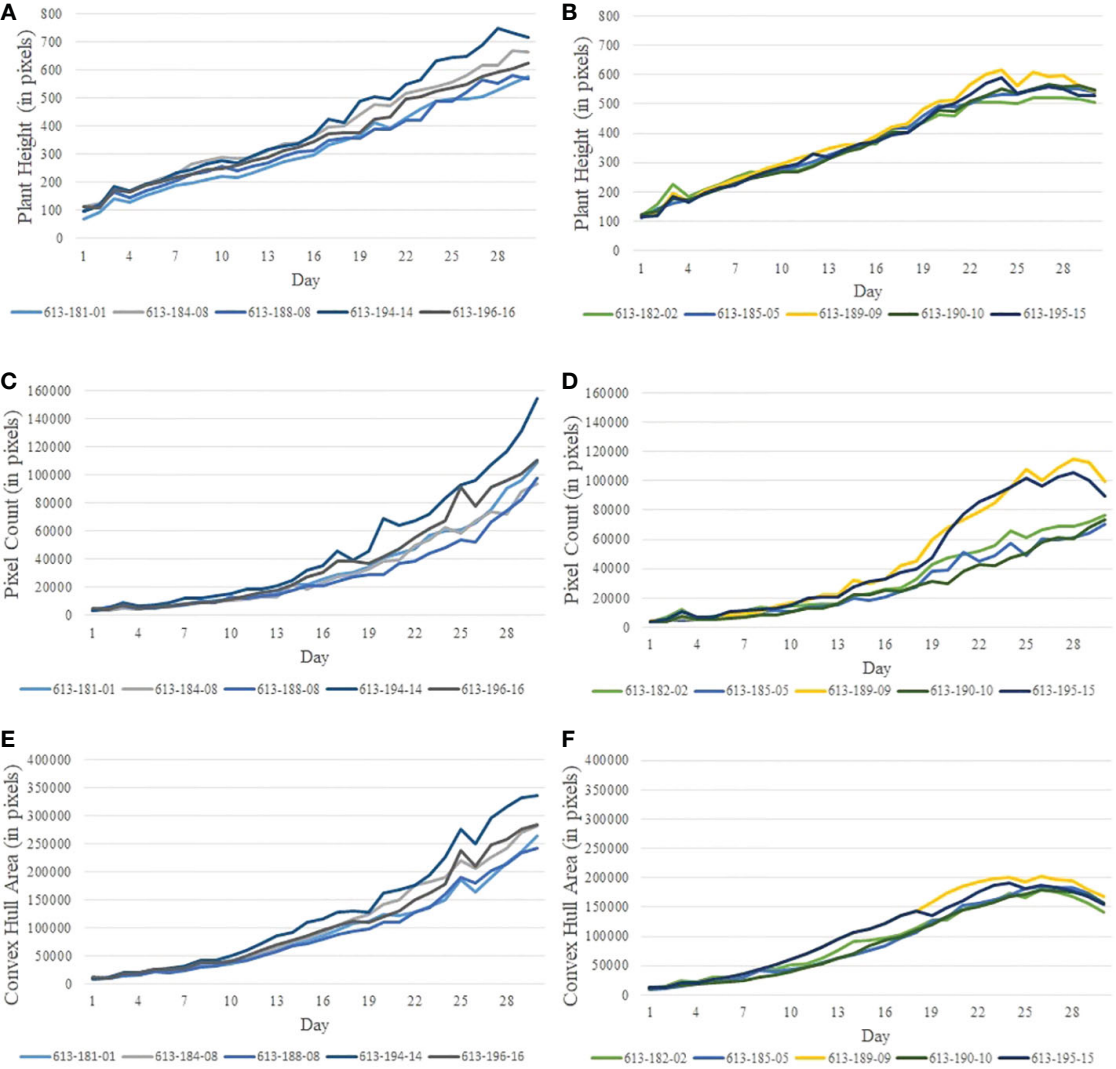
Illustration of nonlinear phenotypic time series using plants from Experiment 2: **(A, B)**- phenotypic time series for the height of plants under the controlled environment and subjected to drought stress, respectively; **(C, D)**- phenotypic time series for plant biomass (measured by pixel count) under the controlled environment and subjected to drought stress, respectively; and **(E, F)**- phenotypic time series for plant size (measured by the area of convex hull) under the controlled environment and subjected to drought stress, respectively.

**Figures 2A, B** show the image sequences of cotton plants enclosed by bounding rectangle and convex hull under normal condition and drought stress, respectively. **Figures 3A, B** show the nonlinear phenotypic time series of the plant height for a set of controlled and drought-stressed plants, respectively. Similarly, **Figures 3C, D** show the nonlinear phenotypic time series for plant biomass (measured by the total number of plant pixels as the function of time) for a set of controlled and drought-stressed plants, respectively. **Figures 3E, F** show the nonlinear phenotypic time series for plant size (measured by the area of the convex hull enclosing the plant) for a set of controlled and drought-stressed plants, respectively.

**Figure 4:**
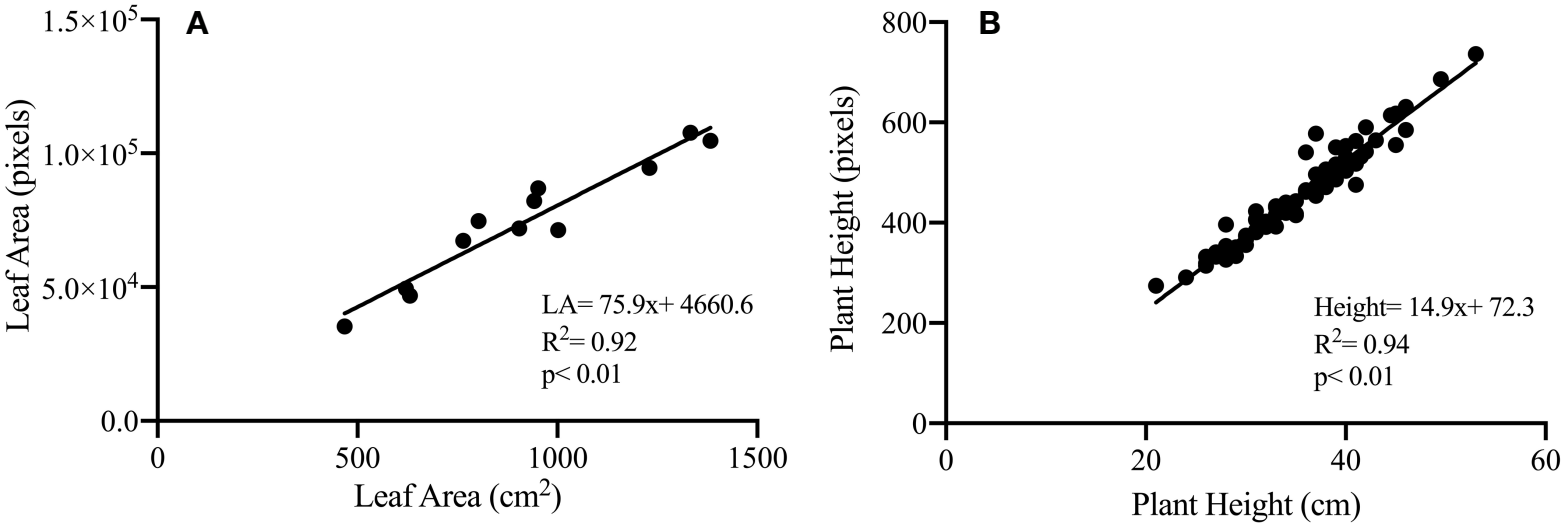
Illustration of correlation between phenotypic traits measured destructively and based on visible light image analysis: projected leaf area (*cm* ^2^) measured destructively and plant biomass (total plant pixels) derived from image analysis (*cm* ^2^) **(A)**; plant height (*cm*) measured destructively and plant height (pixels) derived from image analysis **(B)** for control and dry-down groups.

To validate the phenotypic traits measured noninvasively based on analyzing images captured in the HTPP system against the destructive handheld (low-throughput) techniques, we correlated the projected leaf area (pixels) and plant height (pixels), derived from the RGB camera of the HTPP, against values derived from low-throughput destructive methods (**Figure 4**). Image-derived projected plant biomass and plant height were highly and significantly correlated with the measured leaf area (R^2^ = 0.92, p< 0.01) and plant height (R^2^ = 0.94, p< 0.01) respectively, confirming the hypothesis for the HTPP methods' ability to accurately estimate morphological traits.

#### 2.2.2 Time series smoothening

The noise introduced during the binarization process and the natural change of orientation of plants’ leaves results in unevenness in the phenotypic time series, which poses significant challenges to subspace matching based on dynamic time warping. We use a moving average (MA) filter to smooth the time series to address this. The MA filter is the most common filter in digital signal processing to smooth functions. It is effective for time-domain encoded signals due to its simplicity. The high frequencies to be removed can be controlled by the length of the window of the MA filter.

Given a phenotypic time series *x* of length *m* and a window size *N*, the filtered time series *y* is given by Equation 1 as follows:


(1) $$y\left[i\right]=\frac{1}{N}\sum_{j=0}^{N-1}x\left[i+j-1\right],\;1\leq i\leq m-N+1$$

**Figure 5:**
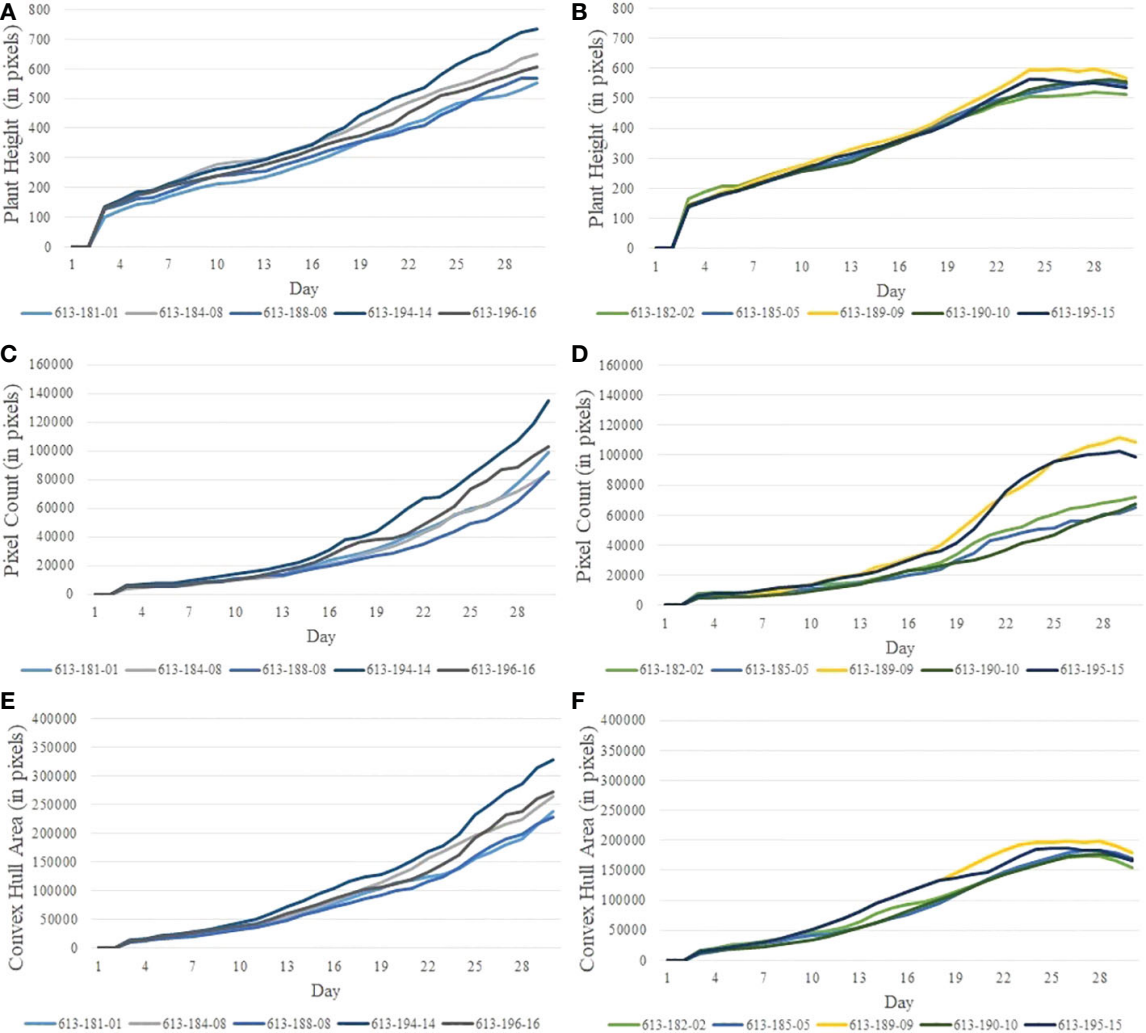
Illustration of smoothened time series using plants from Experiment 2. **(A, B)** plant height for control and stressed plants, respectively; **(C, D)** plant biomass (measured by pixel count) for control and stressed plants, respectively; and **(E, F)** plant size (measured by the area of convex hull enclosing the plant) for control and stressed plants, respectively.

In this paper, a window size of *N*=3 is used for the MA filter. The smoothened time series are used as input to the DTW-based drought stress prediction algorithm. **Figure 5** shows the smoothened time series of the phenotypes (plant height, plant biomass and plant size) for control and stressed plants of Experiment 2.

#### 2.2.3 Stress prediction using DTW

The goal of time series modeling is to study past observations to develop an appropriate model that describes its underlying structure for making predictions. Dynamic time warping (DTW) (Sakoe and Chiba, 1978) is widely used to find the optimal alignment between two given time series. It has been successfully used in automatic speech recognition, gait recognition, and data mining to compare time series with different speeds and deformations. DTW uses dynamic programming to compute a warping function that optimally aligns two time series of variable lengths and measures their similarity. Given two plant phenotypic time series, i.e., *P*=(*P*_1_ *,P*_2_ *,…,P_M_*) and *Q*=(*Q*_1_ *,Q*_2_ *,…,Q_N_*) of respective lengths *M*∈*N* and *N*∈*N*, and *P_i_* and *Q_j_* are the respective phenotypic value on the *i^th^* and *j^th^* days, DTW constructs an *M*×*N* warping path which is a sequence of length *p* of *L* index pairs ((*i*_1_,*j*_1_),(*i*_2_,*j*_2_),…,(*i*_L_,*j*_L_)) and *A*(*P,Q*) is a set of all admissible paths. For a path to be admissible, it should satisfy the following conditions: (a) boundary: *p_1_* =(1,1) and *p_L_* =(*M,N*); (b) monotonicity and all indices should appear at least once: *i_l-1_* ≤*i_l_* ≤*i_l_*_-1_+1 and *j_l-1_* ≤*j_l_* ≤*j_l_*_-1_+1. DTW minimizes the cost of warping *P* and *Q* together, i.e.,


(2) $$DTW\left(P,Q\right)=\underset{p\in A\left(P,Q\right)}{\min}\left({\left(\sum_{\left(i,j\right)\in p}dist\left(P_{i},Q_{j}\right)\right)}^{1/2}\right)$$

Dynamic programming provides an exact solution to the optimization problem at hand. DTW constructs the *M*×*N* matrix of Euclidean distances of corresponding phenotypes where *DTW_i,j_* is the distance between *P*[1:*i*]=(*P*_1_, *P*_2_,…,*P_i_*) and *Q*[1:*j*]=(*Q*_1_, *Q*_2_,…,*Q_i_*) with the best alignment given by the recurrence function given in Equation 3, i.e.,


(3) $$DTW_{i,j}=dist\left(P_{i},Q_{j}\right)+minimum\left(DTW_{i-1,j},DTW_{i,j-1},DTW_{i-1,j-1}\right)$$

where *dist*(*P_i_,Q_j_*)=( *P_i_,Q_j_*)^2^.

Differences in environmental conditions, even in controlled environments, including water content and induced stress, result in variations in the phenotypic sequences for different plants of the same species. However, the plants undergoing stress will have fundamentally different phenotypic trajectories than those growing in normal conditions. Thus, dynamic time warping (DTW) distance is an ideal fit to compare the phenotypic trajectories of plants. The DTW distance between the phenotypic sequences of plants under similar conditions will be significantly different from those of plants under other conditions and can therefore form the basis to differentiate a normal growth sequence from a (drought) stress sequence. Note that all plant image sequences used in this study are of the same length, i.e., *M* = *N*. However, mechanical breakdown or the time-shared based imaging policy in an HTTP often results in the generation of image sequences of unequal lengths. Since DTW effectively compares time series of varying lengths, our proposed VisStressPredict algorithm will be suitable to deal with unforeseeable situations of generating unequal phenotypic time series in any phenotypic measurement environment. This also proves the generalizability of the algorithm.

**Figure 6:**
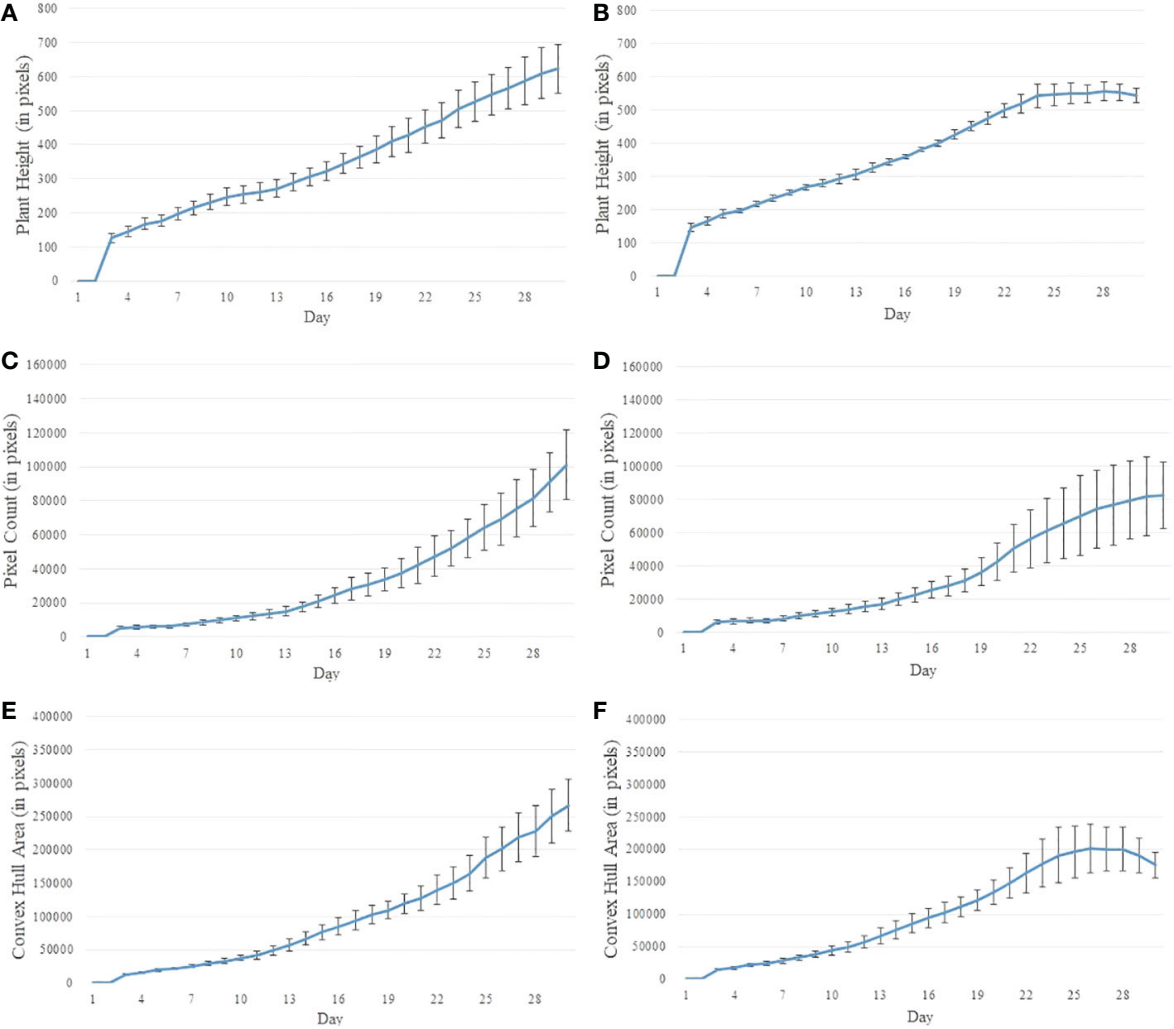
Illustration of representative phenotypic time series with mean and standard deviation using plants from Experiment 2. **(A, B)** plant height for control and stressed plants respectively; **(C, D)** plant biomass for control and stressed plants respectively; and **(E, F)** plant size for control and stressed plants respectively.

In this paper, we propose a DTW-based approach to differentiate between control and stressed plants based on their phenotypic time series. Given a sequence *S* = (*S*_1_,*S*_2,_…,*S_n_*) of length *n* and its subsequence *S_sub_* =(*S*_1_,*S*_2,_…,S*_i_*) of length *i* where 1 ≤ *i* ≤ *n*, we classify the subsequence *S_sub_* as either control or stressed. Two representative sequences *R_c_* and *R_s_* are calculated by element-wise averaging of a set of control and stressed sequences, respectively. **Figures 6A, B** show the representative phenotypic sequences of the height of a plant for control and stressed plants, respectively. **Figures 6C, D** show the representative phenotypic sequences of plant biomass (measured by total plant pixels) for control and stressed plants, respectively. **Figures 6E, F** show the representative phenotypic sequences of plant size (measured by convex-hull area) for control and stressed plants, respectively. The DTW distances *D_c_* and *D_s_* are calculated between *S_sub_* and *R_c_* and between *S_sub_* and *R_s_*, respectively. *D_c_* and *D_s_* are referred to as control DTW distance and stress DTW distance, respectively. The distance is normalized to obtain D*_norm_*, which is then smoothened using a MA filter of window size *N*=6 (Equation 1) to obtain a stress factor, i.e., *SF*.

D*_norm_* is given by:


(4) $$D_{norm}\left(i\right)=\frac{\left|D_{s}\left(i\right)-D_{c}\left(i\right)\right|}{D_{s}\left(i\right)}$$

where *D_s_* (*i*) and *D_c_* (*i*) are given by

*D_s_* (*i*) = *DTW*(*S_sub_,R_s_*) and *D_c_* (*i*) = *DTW*(*S_sub_,R_c_*).

Finally, the stress factor, *SF*, for the subsequence is computed by:


(5) $$SF\left(i\right)=Average\left(D_{norm},i,N\right),$$

where *Average* gives the average of the normalized distances in the window *i*−*N*+1 to *i* or 0. If the stress factor is above a predefined threshold, t^*^, we label that subsequence as stressed. The threshold value t^*^ is defined as:


(6) $$t^{*}=Median\left(Max_{SF}\right)$$

where *Max_SF_* is the set of maximas of the *SF*’s of control plants.

The stress factor threshold t^*^ is taken as the median of the maximas of *SF*’s rather than the mean or maximum is to avoid any outliers in the control set from drastically affecting the threshold t^*^.

Equation 7 gives the conditions for the predicted class.


(7) $$Predicted\;Class\left(i\right)=\{\begin{array}{ll} Stressed & \text{if }SF\left(i\right)\geq t^{*}\\ Control & \text{otherwise} \end{array}$$

Finally, onset of the stress can be determined by identifying the first time stamp in the sequence to have the predicted class to be labeled “Stressed.”


(8) Onset(S)=Θ:∀ 1≤i<Θ PC(i)=Control∧PC(Θ)=Stressed

The proposed method is summarized in **Algorithm 1**.

Algorithm 1 Classify control and stressed sequences and predict onset of stress
**Require:** *S:array*[1…*n*], *S_sub_
*: *S*[1:*i*] 1 ≤ *i*≤ *n,listOfControlSeqs, listOfStressedSeqs* **function** CALCREPRESENTATIVESEQ(*listOfSeqs*)
*
  SumOfSeqs*←*array*[1…*n*]
  **for** each s in listOfSeqs **do
**   **for** i:= 1 to n **do**
     *SumOfSeqs* [*i*]← *SumOfSeqs* [*i*] + *s*[*i*] **   end for
**  **end for
  **
*R_t_
*←*SumOfSeqs/length*(*listOfSeqs*)
  **return** *R_t_
*
**end function
function **VISSTRESSPREDICT(*S, S_sub_
*, *listOfControlSeqs*, *listOfStressedSeqs*)
  *R_c ←_ CALCREPRESENTATIVESEQ(listOfControlSeqs)* 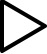 Representative sequence for control
*  R_s ←_ CALCREPRESENTATIVESEQ(listOfStressedSeqs)* 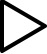 Representative sequence for stress
  *D_norm_
*←*array*[1…*n*]
 * D_norm_MA*←*array*[1…*n*]
  *flag* ← 0
*  day* ← 0
  **for** i = 1 to length(*S*) **do**
    *S_sub_
*← *S*[1:*i*]
    *D_c_
* ← *DTWDistance(R_c_,S_sub_)
    D_s_
* ← *DTWDistance(R_s_,S_sub_)
    D_norm_
*[*i*]← |*D_s_ - D_c_
* |/D_s_
*    D_norm_MA*[*i*]←*Average*(*D_norm_, i, N*)
**    if** *DNormMA*[*i*]>t^*^
 **then**
*     predictedClass* ← “*Stressed*”
**     if** *flag* == 0 **then**
*      firstStressDay ← i
*      *flag* ← 1
**     end if**
    **else**
*     predictedClass* ← “*Control*”
    **end if
   end for
   return** *predictedClass, firstStressDay*
**end function**

### 2.3 HyperStressPropagateNet: Deep neural network based temporal stress propagation using hyperspectral imagery

A hyperspectral image can be represented by a three-dimensional array of intensities, *H*(*x*,*y*,λ), where (*x*,*y*) represents the location of a pixel and λ denotes the wavelength. Thus, it is often referred to as a hyperspectral cube. Intensities at a given wavelength can be represented as a two-dimensional image, and intensity information at a specific location for all wavelengths can be represented by a spectral reflectance curve.

#### 2.3.1 Segmentation

**Figure 7:**
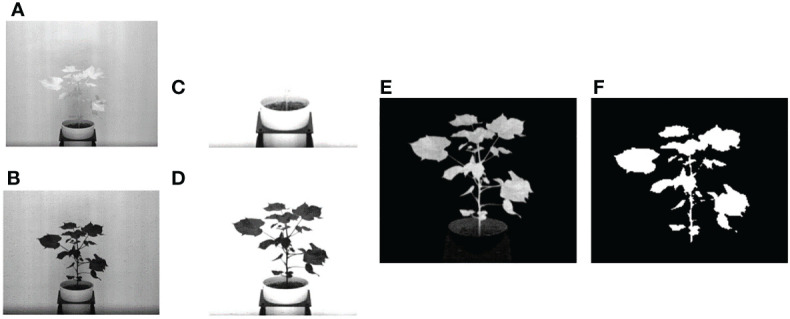
Illustration of spectral band difference based segmentation: **(A, B)** - hyperspectral images of a cotton plant at wavelengths 770 nm and 680 nm, respectively; **(C, D)** - corresponding enhanced images; **(E)** image obtained after subtracting **(C)** from **(D)**; and **(F)** binary image.


We use a spectral band difference-based segmentation approach to create the mask of the plant for subsequent analysis. This segmentation method is useful and efficient for plant phenotyping analysis using hyperspectral or multispectral imagery, since the goal is to analyze only the plant ignoring the background. The segmentation process is illustrated in **Figure 7**. In this approach, two bands of specific wavelengths that have significant contrast in intensity are first identified (**Figures 7A, B**), then enhanced by multiplying a constant factor (**Figures 7C, D**), and finally subtracted from each other to isolate the plant pixels, i.e., the foreground (**Figure 7E**). Based on empirical analysis, the two wavelengths that are effective are 770 nm and 680 nm, and the constant factor is 2. Thus, the enhanced foreground image, (*I_f_*), is given by:

(9) $$I_{f}=2*I_{770}-2*I_{680},$$

where *I*_770_ and *I*_680_ are the images at 770 nm and 680 nm wavelengths, respectively. The enhanced foreground image is then binarized using Otsu's automatic thresholding technique (Otsu, 1979) to generate a binary mask for the plant (**Figure 7F**), which is then used to segment the plant in all bands of a hyperspectral cube for subsequent analysis. Otsu’s method chooses a global threshold so as to maximize the separability of the resultant classes in gray levels. This threshold is then used to convert a grayscale image to a binary image. In this paper, we used graythresh() function of Matlab to generate the global threshold followed by imbinarize() to create the binary mask.

#### 2.3.2 Hyper-pixel generation

**Figure 8:**
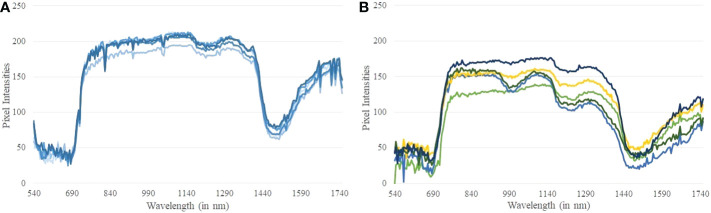
**(A)** Reflectance spectra generated at random pixels of a controlled plant; and **(B)** Reflectance spectra generated at random pixels of a stressed plant.

A hyper-pixel (HP) is defined as HP = {*P*_410,…_ *P*_800_}, where *P_i_* denotes a plant pixel at the wavelength *i*. A reflectance spectrum is generated at each hyper-pixel by plotting the grayscale value of the hyper-pixel over the wavelength range. **Figures 8A, B** show the reflectance spectra generated at randomly selected pixels from a controlled and a stressed plant, respectively. Stomatal response, reactive oxygen species scavenging, metabolic rate, water absorption, and photosynthetic capability are all affected when plants are subjected to drought stress. These collective responses lead to an adjustment in the growth rate of plants as an adaptive response for survival (Osakabe et al., 2014). This phenomenon creates differences in the reflectance spectra at different wavelength ranges generated from hyperspectral imagery of the stressed and controlled plants. It is seen from **Figure 8A** that the reflectance spectra from the controlled plant are very similar. The comparatively dispersed nature of the reflectance spectra of the stressed plant (**Figure 8B**) can be attributed to the varying stress at different parts of the plant. We observe a relatively sharp dip in the reflectance spectra of the stressed plant compared to the controlled plant approximately in the wavelength range of 1200−1300 nm. The difference in the reflectance spectra between the controlled and the stressed plant forms the basis of this algorithm. Note that a sharp decrease in reflectance between 1400−1600 nm wavelength range is guided by the physiological characteristics of the plants. This wavelength range is known for atmospheric water absorption, and is sensitive to vapor reflectance. In this range, light absorption by the plants is significantly high resulting in low gray-scale values in their hyperspectral imagery.

#### 2.3.3 Training and classification

**Figure 9:**
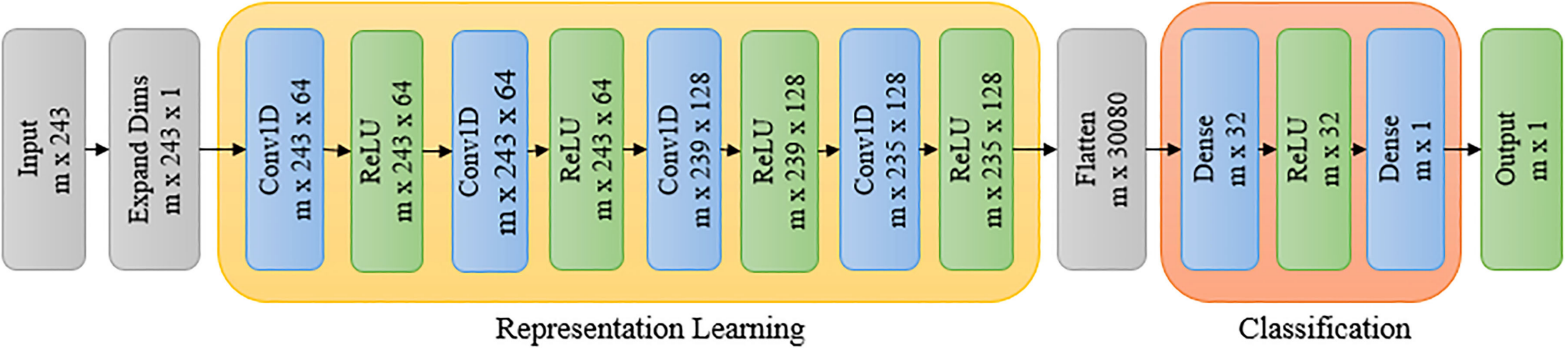
Deep learning architecture for classification of stressed and unstressed pixels.

Convolutional neural network (CNN) models have been effective in various computer vision applications, including segmentation, classification, object recognition, biometrics, and medical imaging (LeCun and Bengio, 1995; Kolhar and Jagtap, 2021). Recently, 1-dimensional (1D) CNNs have been used in natural language processing, speech recognition, and biomedical signal processing where they can perform feature extraction and classification tasks in a single end-to-end model (Jang et al., 2020). In this paper, we use a 1D CNN to classify the reflectance spectra into two classes, i.e., stressed and unstressed. These convolutional layers learn from the representation learning component. Each convolutional layer consists of multiple (N) filters. Each filter of the convolutional layer learns a different feature. The goal of representation learning is to learn the different features in the convolution layers and then use them in the subsequent dense layers for the final classification. The architecture of the proposed network is shown in **Figure 9**. The proposed network consists of two components: representation learning and classification. The details of the network architecture are given below.

The representation learning component consists of four 1D convolutional layers. The size of the input vector is (*m*, 243), where *m* is the number of training examples, each consisting of 243 reflectance values corresponding to a reflectance spectrum. The dimension of this input vector is increased to (*m*, 243, 1) to feed it into a 1D convolutional layer. The 1D CNN layer is followed by a rectified linear unit (ReLU) activation function. There are four such successive 1D CNN layers with ReLU activation. They each have a kernel size of 5 and a stride of 1. The first two convolutional layers have 64 filters, and the ‘same’ padding is used while the last two convolutional layers have 128 filters with the ‘valid’ padding.

The feature vectors obtained after the convolutions are fed to the classification component, which consists of two dense layers. First, the output of the convolutional step, which is a vector of size (*m* × 235 × 128), is ‘flattened’ to a vector of size (*m*, 30080). The flattened vector is then fed to a dense layer in the classification component, which has 32 filters with ReLU activation. The (*m*, 32) vector thus obtained from the dense layer is passed to another dense layer with a sigmoid activation for binary classification between stressed and unstressed classes. The groundtruth for training is developed based on visual inspection of the RGB images of the plants. Drooping can be seen in plants in dry down stage for the last three days of the experiment. The hyperpixels for these last three days are labeled as stressed. The hyperpixels of the plants for all days under the controlled environment are marked as unstressed for the purpose of groundtruth generation. The labeled dataset is split into training, validation, and test sets in the ratio of 0.64, 0.16, and 0.20.

#### 2.3.4 Evaluation metrics

HyperStressPropagateNet has been evaluated using a confusion matrix, precision-recall curve, and *F*_1_-score. These metrics are defined as follows:

- Confusion matrix is a specific tabular representation that allows the visualization of the performance of an algorithm, and is extensively used in the case of statistical classification problems. For a confusion matrix *C*, *C_i,j_* is equal to the number of observations known to be in class *i* but predicted to be in class *j*. Thus, *C*_0,0_ is the true negatives (*T_N_*), *C*_1,0_ is the false negatives (*F_N_*), *C*_0,1_ is the false positives (*F_P_*), and *C*_1,1_ is the true positives (*T_P_*).
- F1-Score is the harmonic mean of precision and recall. The range for F1-Score is [0, 1], with 0 being the worst and 1 being the best prediction. It is defined as:

(10) $$F1-Score=\frac{2\times TP}{2\times TP+FP+FN}$$

Precision (*P*) is defined as *T_P_*/(*T_P_* +*F_P_*) and recall (*R*) is defined as *T_P_*/(*T_P_* +*F_N_*). *F*_1_-score is a better measure than accuracy for unbalanced datasets.

## 3 Experimental results

### 3.1 VisStressPredict: DTW based stress prediction using visible light imagery

**Figure 10:**
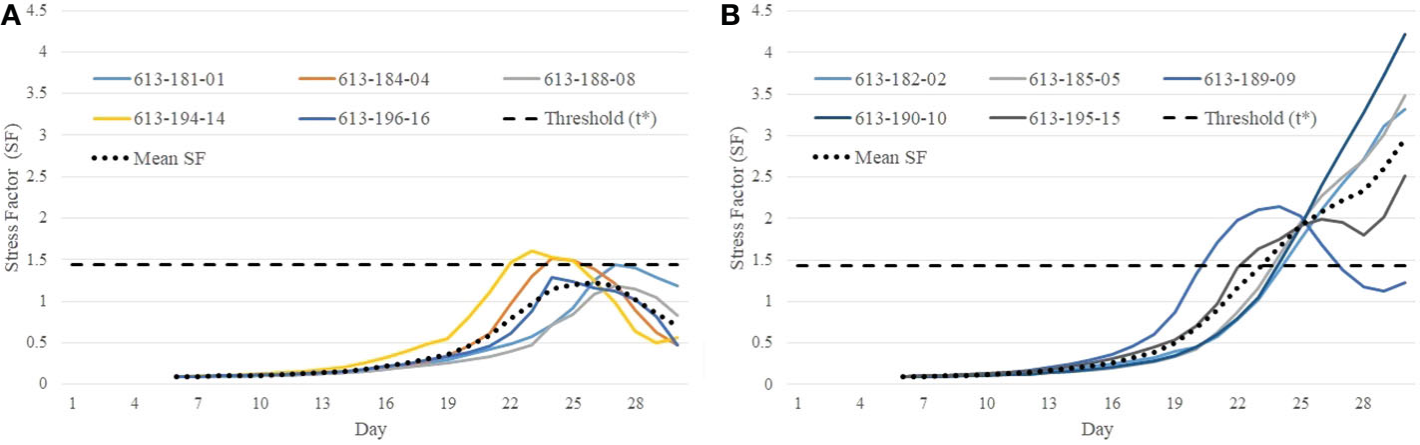
Illustration of difference in behavioral characteristics between control and stressed plants in terms of stress factor curves: **(A)** stress factor curves for control plants; and **(B)** stress factor curves for stressed plants.

The stress factor (*SF*) for each plant is calculated using Equation 5. If the stress factor (*SF*) for a particular plant on a certain day crosses the threshold t^*^, it is predicted to be stressed from that day. The predicted class and onset of stress are given by Equations 7 and 8, respectively. **Figures 10A, B** show the stress factor as a function of time (called as a stress factor curve) for a set of normal and stressed plants, respectively. The figures show that the plants demonstrate similar group behavior. The stress factor curves for normal plants gradually increase, peak around the threshold t^*^, and then gradually decrease. The stress factor curves for the stressed plants, on the other hand, generally keep increasing for the duration of the study.

It is seen from **Figure 10B** that the stress factor curves for stressed plants with plant IDs 613-182-02, 613-185-05, 613-190-10, and 613-195-15 cross the threshold t^*^ on Day 24, Day 23, Day 23, and Day 22, respectively, whereas stress factor curves for the control plants remain below the threshold during the course of the study (**Figure 10A**). The only exception is a stressed plant (ID 613-189-09) in **Figure 10B** whose stress factor curve keeps decreasing after it reaches a peak. This is likely an outlier and may be caused due to imaging artifacts or an anomaly in the computation of the convex-hull area due to plant rotation. These defects may occur in plant images and can be fixed with a more rigorous image processing and correction pipeline.

### 3.2 HyperStressPropagateNet: Deep neural network based temporal stress propagation using hyperspectral imagery

**Figure 11:**
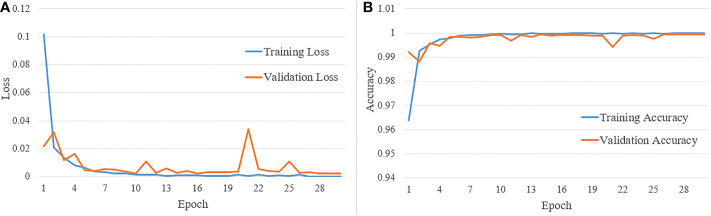
**(A)** Training and validation loss vs number of epochs; and **(B)** training and validation accuracy vs number of epochs.

**Figure 11A** shows the training and validation loss versus the number of epochs, and **Figure 11B** shows the training and validation accuracy versus the number of epochs. The total number of epochs used during training is 30. From the two sets of graphs, it is evident that the validation loss and accuracy closely follow the training loss and accuracy, respectively. Also, the model converges, and validation accuracy reaches above 95% within 10 epochs.

**Figure 12:**
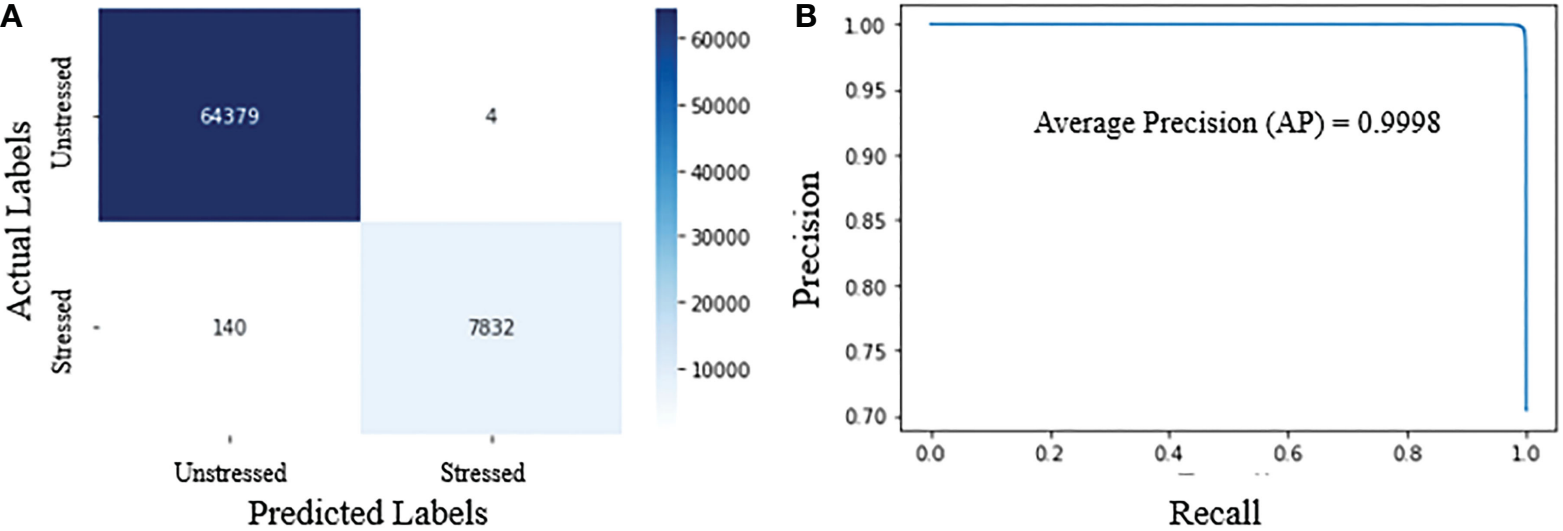
Performance metrics for HyperStressPropagateNet: **(A)** confusion matrix; and **(B)** precision-recall curve.

**Figure 12A** shows the confusion matrix, demonstrating the accuracy of classifying hyperpixels into stressed and unstressed classes. The confusion matrix in **Figure 12A** shows that at a threshold probability of 0.5, the false positives and false negatives are extremely low. Precision and recall for our proposed classifier are 0.99 and 0.98, respectively. *F*_1_-score is 0.98. The very high values for precision, recall, and *F*_1_-score show that the model can accurately distinguish between stressed and unstressed spectra.

**Figure 12B** shows the precision-recall curve for different thresholds for the predicted probabilities. The model outputs the probabilities of pixels being stressed from which the predictions are obtained using a threshold. This threshold is generally kept as 0.5. As the threshold is increased from 0 to 1.0, the predictions obtained from the probabilities vary, and so do the precision and recall values. The model with the highest area under the precision-recall curve is generally deemed optional. **Figure 12B** shows that the precision and recall values are very high for the entire range of threshold for the proposed model, thus giving a very high area under the precision-recall curve close to 1.0. The average precision for the model is also very high, i.e., 0.9998. The various performance metrics demonstrate the efficacy of the proposed algorithm.

**Figure 13:**
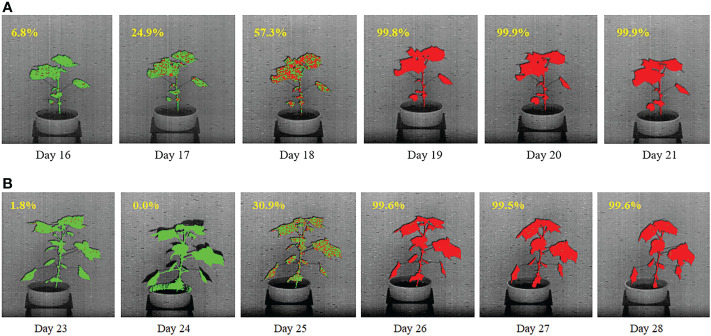
Illustration of qualitative and quantitative temporal propagation of stress using **(A)** a plant from DD1 group and **(B)** a plant from DD2 group. The percentage of stress pixels are shown at the top-left corner of each image.

**Figures 13A, B** show the temporal propagation of stress using hyperspectral image sequences of cotton plants from Experiment 1 (Plant ID: 613-200-20) and Experiment 2 (Plant ID: 613-195-15), respectively. In this figure, the hyperpixels classified as stressed and unstressed are shown in red and green, respectively, for qualitative visualization of temporal stress propagation. The percentage of the stressed pixels to the total plant pixels is shown at the top left of each image.

For the plant in **Figure 13A**, drought stress was introduced on Day 13. The figure shows a gradual increase in the stress symptoms that started on Day 16, when 6.8% of the plant is labeled as stressed (marked in red). The percentage of stress pixels increased to 24.9% on Day 17 and 57.3% on Day 18. There is almost no green pixel present in the plant on the last two days, i.e. on Day 20 and Day 21, which implies that the whole plant is stressed. For the plant in **Figure 13B**, drought stress was introduced on Day 20. The figure shows that stress symptoms did not appear in the plant until Day 25 when, 30.9% of the plant are labeled as stressed (marked in red). The figure shows that the plant is considerably stressed on Days 26 and 27, with very few unstressed pixels (shown in green). There is almost no green pixel present in the plant on Day 28, which implies that the whole plant is stressed.

**Figure 14:**
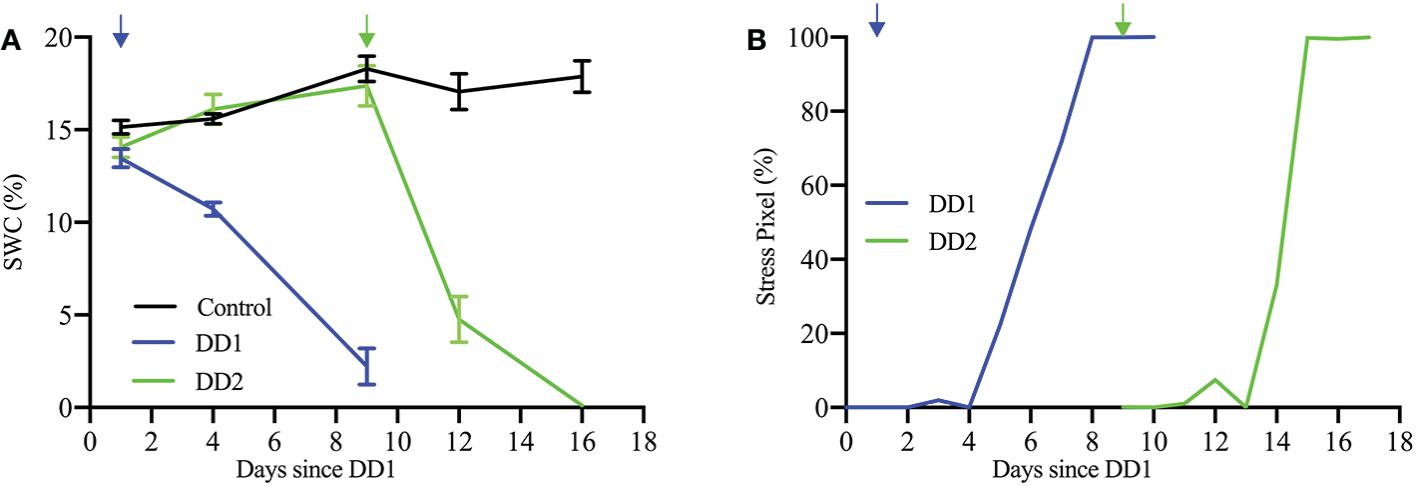
**(A)** SWC (%) for the control and the two dry-down groups (DD1, Plant ID: 613-200-20 and DD2, Plant ID: 613-195-15); and **(B)** stress pixel (%) over days since DD1 for the same plants.

The limited water availability in the soil is confirmed by changes in the soil water content (SWC), as shown in **Figure 14A**. Soil water content was measured using a HH2 type Moisture Meter (Eijkelkamp, NL) connected to a ML3 ThetaProbe Soil Moisture Sensor (Delta-T Devices, UK). Dry-down treatment resulted in a decline of SWC from initial conditions (field capacity ∼ 16% SWC) to 15.6% of field capacity (∼ 2.5% SWC) and 6.3% of field capacity (∼ 1.0% SWC) for DD1 and DD2, respectively, at the end of each dry-down period. **Figure 14A** also indicates the immediate effect in the SWC that follows the cessation of watering. **Figure 14B** shows the temporal progression of percentage of stress pixels for a plant from Experiment 1 (Plant ID: 613-200-20) and a plant from Experiment 2 (Plant ID: 613-195-15). The quantitative visualization of temporal stress propagation of these two plants are shown in **Figures 13A, B**, respectively. The excellent correlation between the SWC and the corresponding temporal progression of the percentage of stress pixels computed by HyperStressPropagateNet for both experiments demonstrates the efficacy of the proposed method.

## 4 Discussion

The paper introduces two novel algorithms to understand the impact of stress on plants. First, an approach to predict the onset of stress in drought-affected plants is presented. The algorithm, named as VisStressPredict, uses an extension of dynamic time warping based on the time-series analysis of plant phenotypes derived from visible light image sequences. The paper also introduces a novel method, i.e., HyperStressPropagateNet, to examine the propagation of stress in plants over time. The deep learning based algorithm uses a convolutional neural network to classify hyperpixels into stressed and unstressed categories. Although both methods have been evaluated using cotton plant image sequences, they can be generalized to any plant species to study the temporal effect of any kind of stress, e.g., thermal and salinity. Thus, the methods have the potential to help differentiate between stress-tolerant and stress-susceptible genotypes for sustainable agriculture. Note that VisStressPredict and HyperStressPropagateNet fundamentally differ in their goals and hence in the input image sequences, underlying approaches, and final outcomes. VisStressPredict identifies the onset of stress on the plant as a whole, but HyperStressPropagateNet maps the stress in a plant at a finer resolution. However, the onset of stress as predicted by VisStressPredict (**Figure 10**) correlates extremely well with the day of appearance of stress pixels in the plants as computed by HyperStressPropagateNet (**Figure 13**). This establishes the dove-tailed relationship between the two proposed algorithms.

The efficacy of the proposed algorithms depends on the reliability of the phenotypes. The accuracy of the computed phenotypic time series depends on many factors, including accuracy in image segmentation, the effectiveness of denoising, and the stability of the plant structure derived from images. The phenotypes such as plant height, plant area, and convex-hull area are derived from the RGB images through a series of image processing steps (See Section 2.2.1). Segmentation of the plant is the basis for image-based phenotypic computation, and inaccuracies in segmentation will result in imprecise computation of plant phenotypes, including its height and convex-hull area (Das Choudhury, 2020a). In addition, inherent challenges introduced while imaging the plant, such as those due to plant rotation, also impact the accuracy of some phenotypes, including plant area and convex-hull area (Bashyam et al., 2021). The plant rotation may cause shrinking of the convex-hull area computed from the imagery from the previous day, although the plant has grown bigger (Maddonni et al., 2002; Das Choudhury et al., 2016). All these factors result in unevenness in the phenotypic time series (**Figure 3**). This unevenness affects the performance of the subsequence-based DTW matching, which explains the outlier stress factor curve (plant ID 613-189-09) in **Figure 10B**. The impact of the error may be ameliorated to some extent by smoothening, as explained in Section 2.2.2.

Finally, it is worth noting that even with a very limited number of stress days in the dataset, the proposed VisStressPredict algorithm shows excellent performance as expressed by the empirically determined stress factor. The mean stress factor curve in **Figure 10A** remains below the threshold during the course of the study for the control plants, whereas, it crosses the threshold on Day 23 and keeps on increasing during the rest of the days for the stressed plants (**Figure 10B**). The method’s potential to predict stress, even in its early stages, demonstrates its efficacy. However, in future work, we will explore the generality of the method by examining the performance of the algorithms on a large dataset with different plant species where plants are subjected to stress for a longer duration.

The dataset used in the study consists of images of cotton plants that are visibly drooped (but not visibly dried as seen by a change of color) under stress. Thus, it is not possible to quantify the stress at the fine pixel scale based on analyzing color features using visible light images. The hyperspectral image analysis for temporal stress propagation achieves the novel objective of identifying the stress location in the plant before the visible stress symptoms appear in the plant. Our study shows an excellent correlation between the soil water content and the percentage of stress pixels in the plants (**Figure 14**). The figure shows that as the soil water content decreases, the stress in plants increases. The Pearson correlation coefficients calculated for SWC and stress pixel percentage for the said plants from the two dry-down groups (DD1, Plant ID: 613-200-20 and DD2, Plant ID: 613-195-15) are -0.972 and -0.735, respectively. The early detection of stress susceptibility acts as an alarm to the deteriorating plant health, and appropriate intervention, e.g., adequate watering of the plant, may help recover the plant’s health. Future work will consider the identification of wavelengths that carry the most salient information on drought stress prior to the classification for improved computational complexity.

## 5 Conclusion

The paper introduces two novel algorithms, i.e., VisStressPredict and HyperStressPropagateNet, to study stress response in plants in greater spatial and temporal resolution by analyzing visible light and hyperspectral imagery. While RGB cameras capture the visible part of the light spectrum in only three broad bands (red, green, and blue), hyperspectral cameras typically capture a broad range of wavelengths at very narrow intervals of a few nanometers. The VisStressPredict algorithm predicts the onset of stress in plants using an enhanced dynamic time warping approach from the phenotypic time series derived from visible light images. The HyperStressPropagateNet algorithm, in contrast, identifies the location of stress in the plants using a deep learning approach from the hyperspectral imagery. The algorithm has been used to illustrate the temporal propagation of stress both qualitatively and quantitatively. The efficacy of the two algorithms is demonstrated using a set of control and drought-stressed cotton plants imaged in an HTTP system. Both the algorithms have the potential to examine the response to other kinds of biotic and abiotic stresses in plants, and can be applied to any kind of plant species.

## Data availability statement

The raw data supporting the conclusions of this article will be made available by the authors, without undue reservation.

## Author contributions

SD conceived the idea, developed and implemented the algorithm, conducted experimental analysis, led the manuscript writing and supervised the research. SS developed and implemented the algorithm, conducted experimental analysis and contributed to the manuscript writing. AM led the dataset design, performed validation experiments and contributed to the manuscript writing. AS and TA critically reviewed the manuscript and provided constructive feedback throughout the process. All authors contributed to the article and approved the submitted version.

## Footnotes

1
https://www.alliedvision.com/en/support/technical-documentation/prosilica-gt-documentation

2
https://www.manualslib.com/products/Headwall-Hyperspec-Inspector-10421605.html
